# Multi-task approach based on combined CNN-transformer for efficient segmentation and classification of breast tumors in ultrasound images

**DOI:** 10.1186/s42492-024-00155-w

**Published:** 2024-01-26

**Authors:** Jaouad Tagnamas, Hiba Ramadan, Ali Yahyaouy, Hamid Tairi

**Affiliations:** Department of Informatics, Faculty of Sciences Dhar El Mahraz, University of Sidi Mohamed Ben Abdellah, 30000 Fez, Morocco

**Keywords:** Breast Ultrasound segmentation and classification, Breast tumors, Convolutional Neural Networks, Self-Attention, MLP-Mixer, Channel Attention

## Abstract

Nowadays, inspired by the great success of Transformers in Natural Language Processing, many applications of Vision Transformers (ViTs) have been investigated in the field of medical image analysis including breast ultrasound (BUS) image segmentation and classification. In this paper, we propose an efficient multi-task framework to segment and classify tumors in BUS images using hybrid convolutional neural networks (CNNs)-ViTs architecture and Multi-Perceptron (MLP)-Mixer. The proposed method uses a two-encoder architecture with EfficientNetV2 backbone and an adapted ViT encoder to extract tumor regions in BUS images. The self-attention (SA) mechanism in the Transformer encoder allows capturing a wide range of high-level and complex features while the EfficientNetV2 encoder preserves local information in image. To fusion the extracted features, a Channel Attention Fusion (CAF) module is introduced. The CAF module selectively emphasizes important features from both encoders, improving the integration of high-level and local information. The resulting feature maps are reconstructed to obtain the segmentation maps using a decoder. Then, our method classifies the segmented tumor regions into benign and malignant using a simple and efficient classifier based on MLP-Mixer, that is applied for the first time, to the best of our knowledge, for the task of lesion classification in BUS images. Experimental results illustrate the outperformance of our framework compared to recent works for the task of segmentation by producing 83.42% in terms of Dice coefficient as well as for the classification with 86% in terms of accuracy.

## Introduction

Breast cancer is considered the most common cancer and the second leading cause of cancer-related mortality in women [1]. The International Agency for Research on Cancer performed a study [2] and found that nearly 2.1 million new breast cancer cases and over half a million new deaths were reported globally during 2018. Breast ultrasound (BUS) imaging is emerging as a complementary screening method for women and can be used as a diagnostic method for breast cancer [3]. Early detection and diagnosis of breast tumors can reduce the mortality rate. Therefore, BUS remains a cheap and safe technique that can be executed using portable devices at the patient’s bedside and is accessible globally [4]. However, diagnosis using BUS requires probes that depend heavily on the operator [5]. Moreover, two or three volumes are acquired for each breast per examination, which results in radiologists and clinicians spending an inordinate amount of time reviewing large volumes of BUS images and making accurate disease diagnoses [6]. In addition, handheld probes are highly sensitive instruments, which makes them susceptible to capturing noise in addition to the ultrasonic images; consequently, it is difficult to properly perform the diagnosis process [7]. Hence, there is an urgent need to develop structured and intelligent systems to help medical professionals diagnose breast tumors with high accuracy. However, developing such systems is challenging because of the high similarity between benign and malignant lesions, irregular tumor boundaries, and the various sizes of shapes and sized for lesions.

Deep learning algorithms have recently been applied in several research domains including the medical imaging field. In recent years, computer vision tasks such as segmentation, classification, and detection have been performed using convolutional neural networks (CNNs), where they have obtained state of the art (SOTA) results and remain the most commonly used networks in medical imaging analysis applications, particularly UNet architectures [8]. Despite their popularity, the primary limitation of CNNs is that they learn information from images using localized receptive fields, which causes their learning capabilities to fail when capturing long-range dependencies [9]. Owing to the great success of transformers [10] in natural language processing, great attention has been paid to self-attention (SA) mechanism-based architectures in many computer vision tasks [11] to improve their nonlocal modeling capability, as they are not subject to the limitations of CNN architectures [12].

Recently, vision transformer (ViT), which is a transformer for vision applications [13], have been investigated in medical image analysis and have achieved SOTA results for many tasks including organ and tumor segmentation as well as disease detection and classification [14]. For medical image segmentation, two designs that employ transformers have been proposed in literature: pure transformers-based models and hybrid models. The first category is U-shaped models built upon ViTs or its variants without any convolutional layers. This allows the learning of long-range semantic information, in contrast to CNN-based architectures. An example is Swin-Unet [15], which is a purely transformer-based method. The second family is hybrid models that modify the encoder-decoder architecture by replacing either the encoder or decoder module with a transformer [16]. An example is TransUNet [17], which has demonstrated good performance because its ability to capture long-range dependency owing to the SA mechanism of transformers, as well as preserving low-level details owing to the intrinsic locality of the convolution operations.

In addition to medical image segmentation, another challenging problem in medical imaging is classifying input images or regions of interest (ROI) in these images into meaningful categories. In addition to CNNs, ViTs have been successfully applied to medical image recognition and classification [14]; and a competitive alternative called multilayer perceptron (MLP)-Mixer has been proposed by Tolstikhin et al. [18] to perform image classification using exclusive MLPs without convolutions or attention blocks. The experiments reported in ref. [18] demonstrated that MLP-Mixer is built upon a simple architecture and produces comparable results to SOTA classifiers, while achieving a good compromise between the accuracy and computational resources required for training and inference.

Motivated by the great success of ViTs in medical image analysis and in particular in the task of segmentation and classification, and inspired by the works in TransUNet [17] and MLP-Mixer [18], we propose an efficient multi-task framework that performs sequential BUS tumor segmentation and tumor type classification. Our framework contains two main steps: first, segmentation of the tumor region, which helps in focusing only on the features of that part of the image; and second, classification of the extracted lesion region into two classes: malignant and benign. To perform the segmentation task, which was inspired by TransUNet [17], we propose an encoder-decoder-based model using a modified U-Net architecture. The encoder is composed of two encoders, where the images are passed in parallel to both efficientNetV2 [19] and an adapted ViTs encoder to extract enriched features and context information at different scales. To combine the feature maps extracted by both the encoders, we design a channel attention fusion (CAF) module that incorporates a squeeze-and-excitation (SE) block [20] for channel attention. The SE block selectively emphasizes the informative features from both encoders, facilitating the integration of high-level and local information. The attention mechanism within the CAF module enables effective feature combinations. The combined features using the CAF module constitute the input to the decoder, where the mask image is reconstructed using skip connections from the efficientNetV2 encoder. In addition to the power of the ViTs encoder, we opted to use efficientNetV2 [19] instead of a CNN encoder, because efficientNetV2 uses a technique called compound scaling to increase the depth, width, and resolution of the network in a balanced manner. This allows efficientNetV2 to capture more context from the image and produce more accurate segmentation, and it is designed in a way that uses fewer parameters and fewer computational resources as compared to other networks. In the second step, a robust tumor classifier is proposed by testing both the ViTs architecture and MLP-Mixer model. ViTs are exploited to leverage the capabilities of the SA mechanism for accurate detection of BUS tumors. In addition, we explore a pre-trained MLP-Mixer model that depends solely on MLPs to classify segmented tumor regions. We investigate the performance of the latter model in comparison with that of the ViT model. Specifically, we fine-tune the BUSI dataset [21] on the pre-trained models of both ViTs and MLP-Mixer after segmenting the lesion regions from the images. The results obtained underscore the great capabilities of classifying the images when using the ViT architecture, depending on the attention mechanism, or the MLP-Mixer relying exclusively on MLPs. In summary, the proposed method contributes to literature as follows:

1. We propose an efficient multi-task framework for BUS segmentation and classification. We leverage the strengths of both efficientNetV2 and adapted ViTs encoders, extracting enriched features and context information at different scales.
2. We design a CAF module based on the SE block for effective feature combination between the dual encoders. Specifically, the module selectively emphasizes important features from both encoders, improving the integration of high-level and local information.
3. We leverage the MLP-Mixer model that depends solely on MLPs to perform BUS images classification. The latter is being used for the first time for this task, to the best of our knowledge. We demonstrate the capabilities of both ViTs and MLP-Mixer in accurately classifying BUS images, with ViTs relying on attention mechanisms and MLP-Mixer relying exclusively on MLPs.

Computer-aided diagnosis (CAD) systems are increasingly utilized to aid healthcare professionals in diagnosing various diseases and cancers, including breast cancer. Tasks such as the detection, segmentation, and classification of tumor regions in BUS are largely addressed in CAD systems.

### BUS segmentation

Previous studies have described various CNN-based methods for breast mass segmentation. Vigil et al. [22] presented an architecture based on a deep convolutional autoencoder to extract latent-space features for segmenting BUS images. Xing et al. [23] utilized a generative adversarial network (GAN) and a CNN based on ResNet [24] as generators for tumor region segmentation to form a semi-pixel-wise cycle model. Singh et al. [25] segmented BUS tumors using a context-aware network based on atrous convolutions, where GAN was utilized to evaluate the performance of the segmentation. Lei et al. [26] proposed a network that performs boundary regularization to segment BUS images. In addition, Lei et al. [27] improved the segmentation of breast structure results using the self-co-attention technique. Kumar et al. [28] introduced a U-shaped architecture called multi-UNet to perform the segmentation of BUS images. In ref. [29], an architecture where attention blocks are incorporated into the U-Net architecture was proposed. Tong et al. [30] introduced a modified U-Net architecture based on a mixed attention loss function to segment BUS tumors. Cao et al. [15] created Swin-UNet by substituting the convolutional encoding and decoding operations of U-Net with a swine transformer module. Chen et al. [17] proposed TransUNet, which implements CNNs to extract features and subsequently feeds them directly to a transformer to capture richer features. Based on the TransUNet backbone, Yang HN and Yang DP [31] combined CNN and swine transformer blocks for feature extraction as an encoder in a pyramid-shaped network for BUS segmentation. Recently, Al-Battal et al. [32] proposed a weakly trained U-shaped segmentation network with an encoder and a multipath decoder, where the latter provides more loss propagation from feature maps to deeper layers and the encoder, as well as efficient upsampling of feature maps that leads to the preservation of high-resolution information. Farooq et al. [33] proposed a semi-supervised mean teacher and student model that utilizes the U-Net model with residual and attention blocks as a backbone network for BUS image segmentation.

### BUS classification

In literature, methods classify BUS images can rely and use the manual extraction of different type of features like shape, texture, lesion borders, margin and orientation. In this context, Moon et al. [34] relied on a mixture of features extracted from ultrasound images, composed of texture, morphological, and descriptor features, to classify tumors. Flores et al. [35] relied on the use of distinct morphological and textual spatial information to perform classification tasks. Similarly, Gómez et al. [36] extracted 22 morphological features after applying the watershed transformation technique to segment BUS images, where feature selection was performed using the minimum-redundancy-maximal-relevance criteria. Tanaka et al. [37] suggested the implementation of a CAD system based on CNNs to differentiate between benign and malignant breast lesions in ultrasound images. The dataset consisted of more than 1000 images, and the reported accuracy of the system was 89%. Han et al. [38] trained GoogleNet [39] using a dataset that included 7408 ultrasound images, consisting of 4254 benign and 3154 malignant lesions, with an accuracy of approximately 0.9, a sensitivity of 0.86, and a specificity of 0.96. Wang et al. [40] suggested a CNN architecture based on a modified Inceptionv3 architecture to effectively extract features from BUS images. Byra et al. [41] used transfer learning to retrain pre-trained models, mainly VGG19, on an ultrasound image dataset after applying the rescaling layer to the image pixels, which aimed to convert them to an RGB representation. Xiao et al. [42] examined the effectiveness of transfer learning using the InceptionV3, Xception, and ResNet50 models on an ultrasound dataset. Ayana and Choe [43] investigated the effectiveness of ViT for classifying BUS images by introducing a novel method for transfer learning. In ref. [44], an architecture (SAFNet) was proposed that combines ResNet-18 and a spatial attention mechanism to form a backbone for feature extraction. Zhong et al. [45] developed a feature fusion network called MsGoF to classify BUS tumors as malignant or benign. Sirjani et al. [46] classified BUS tumors using a modified InceptionV3 network in which they adjusted the number of residual modules and other hyperparameters.

The proposed method bridges a significant gap between existing research methods and introduces contributions. While previous methods for both segmentation and classification predominantly relied on CNNs or transformers for BUS image analysis, the proposed method combines the strengths of both architectures. This integration allows the capture of fine-grained spatial details through CNNs and models the global context and long-range dependencies through transformers. Additionally, the proposed method addresses the limitations of TransUNet, which is one of the few methods that incorporates both CNNs and transformers, by introducing an efficientNetV2 encoder and a CAF module. These enhancements improve the understanding of complex spatial relationships, facilitate effective feature combinations, and enhance segmentation and classification accuracy. Furthermore, the exploration of ViTs and MLP-Mixer models as alternative classification approaches adds novelty, offering insights into the effectiveness of attention mechanisms and MLPs in BUS image classification.

## Methods

**Fig. 1:**
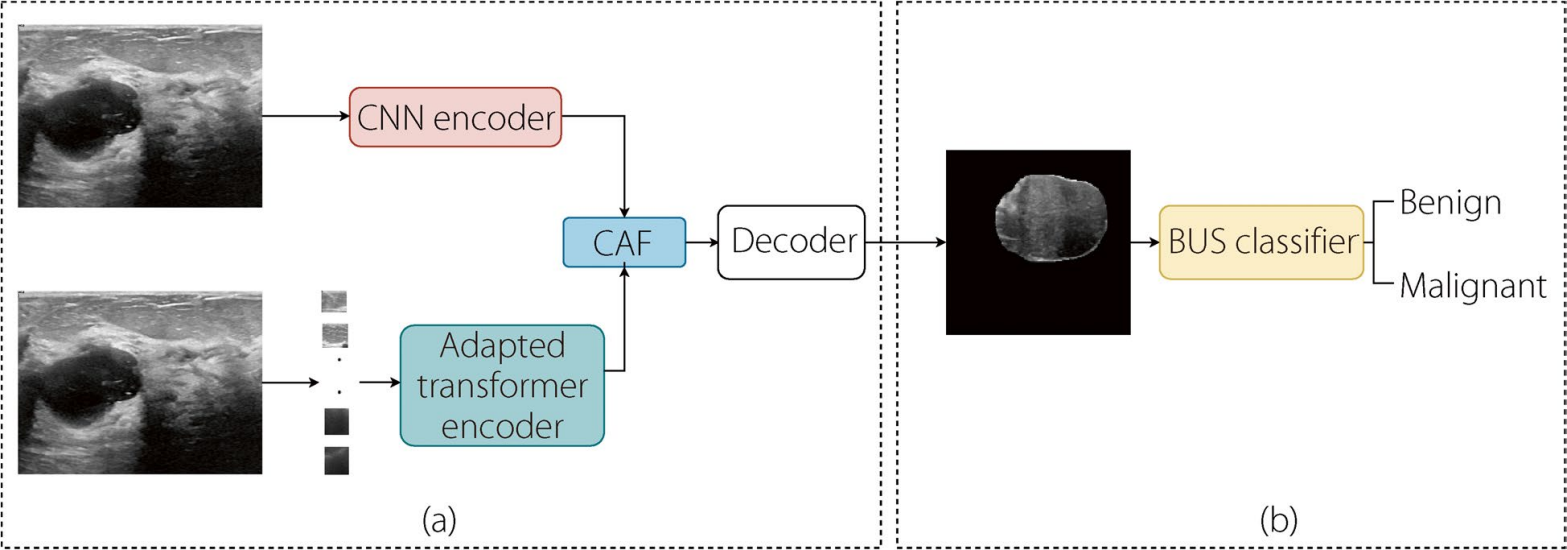
Flow chart of our contribution. (**a**) The proposed architecture to perform the segmentation of BUS images; (**b**) The proposed method to classify the segmented BUS images

In this study, we propose an efficient multi-task framework for segmenting and classifying tumors from BUS images. The proposed approach performs two tasks during two main phases. First, the segmentation architecture was trained on BUS images and their corresponding masks to extract the tumor region from its surroundings in the image. Secondly, the proposed approach enhances its performance by exploiting the potential of transformers, in which an adapted ViT model was employed for the classification of the segmented tumor region. Additionally, we investigated the MLP-Mixer to capitalize on its ability to classify BUS images by relying solely on MLP blocks without the need for high computational resources for training and inference. Figure 1 shows an overview of the proposed framework.

### Segmentation architecture

**Fig. 2:**
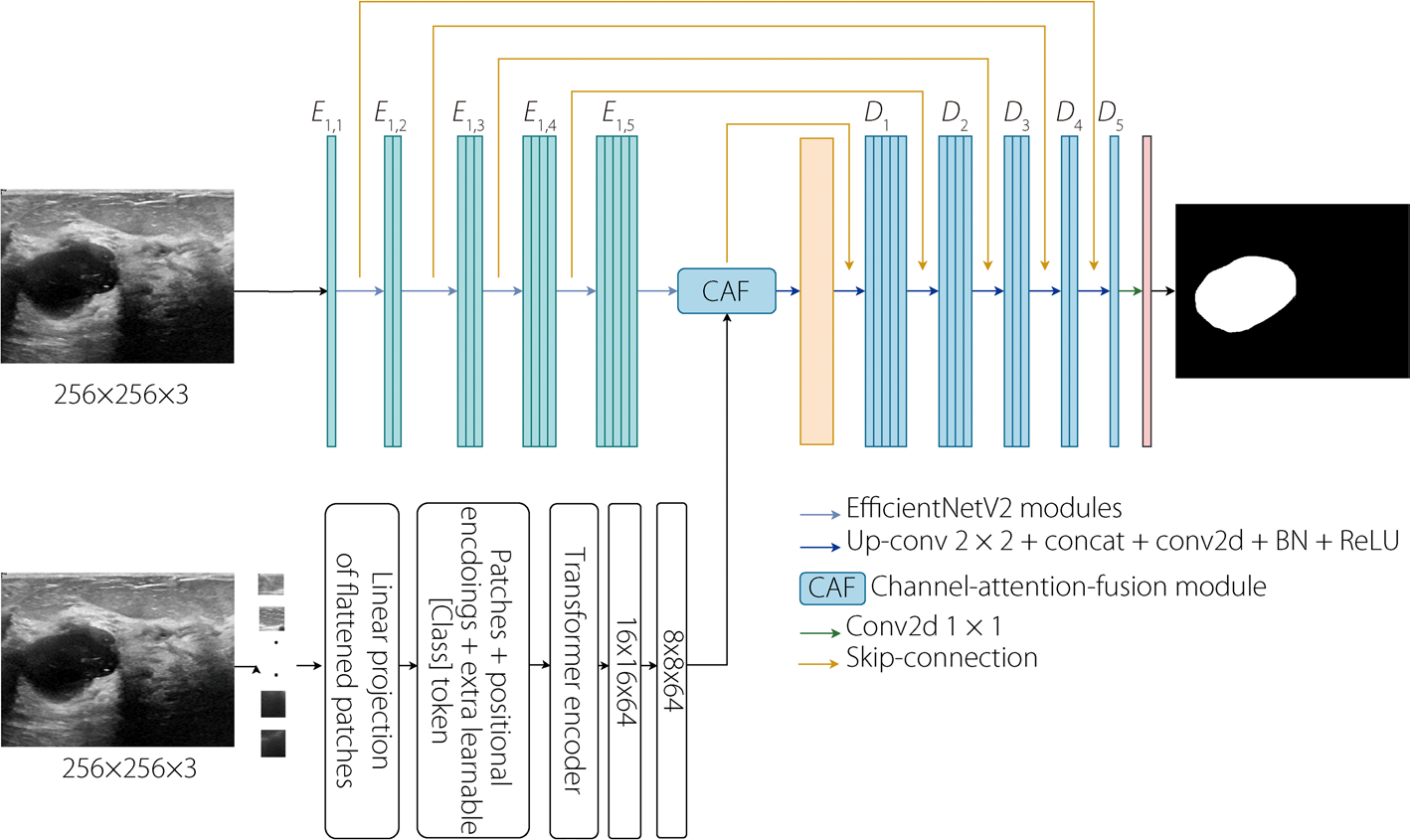
The proposed architecture to perform the segmentation of BUS images

Figure 2 shows the proposed model for the segmentation task, which comprises two parallel encoders, each with distinct characteristics. The first encoder is built on the EfficientNetV2-L backbone, which serves as the foundation for feature extraction from the input image instead of a conventional set of convolution layers. To accomplish this, several layers within the blocks of the backbone are employed as feature extractors to capture diverse aspects of image information. In addition, using EfficientNetV2-L [19] as an encoder allows the network to require fewer computational resources than other commonly used CNN models. Furthermore, EfficientNetV2-L incorporates both MBConv and fused-MBConv, enabling it to capture more diverse and informative feature maps from the images. Therefore, using efficientNet as the backbone of the encoder can lead to more accurate segmentation results, particularly for the studied segmentation tasks. We investigated different combinations of blocks to construct the encoder from EfficientNetV2-L with pre-trained ImageNet [47] weights, where blocks that are closer to the input image tend to capture low-level features, including textures, edges, and patterns, whereas the deep blocks in the network contribute to learning higher-level semantic features. For further details on the advantages of EfficientNetV2 compared to previous CNN architectures, please refer to ref. [19]. Table 1 lists the layers of EfficientNetV2-L, which was used as the backbone of the first encoder. Concurrently, the second encoder operates based on a transformer architecture and functions similar to the original ViT [13] except for the input image resolution, projection dimension, number of multi-head-self-attention (MSA) heads, and number of transformer layers, as shown in Table 2. The encoder extracts global deep features from the input image by leveraging the SA mechanism of the transformer model, thereby providing a complementary representation of the image features. The first and second encoders output feature maps with dimension (8 × 8 × 3840) and $\left(\frac{H}{P}\times \frac{W}{P}\times d\right)$, respectively, where *H*, *W*, and *P* represent the height, width, and patch size of the input image, respectively, and *d* represents the output feature vector.

**Table 1:** EfficientNetV2-L layers used as components for the CNN encoder

Number	Layer	Output shape
1	input 1	256 × 256 × 3
2	block1d_project_activation	128 × 128 × 32
3	block2g_expand_activation	64 × 64 × 256
4	block4a_expand_activation	32 × 32 × 384
5	block6a_expand_conv	16 × 16 × 1344

**Table 2:** Our adapted Transformer parameters used in the encoder

ViT version	Image resolution	Projection dimension	Number of MSA heads	Number of transformers layers
ViT-base	224 × 224	768	12	12
Our adapted transformer	256 × 256	64	8	12

**Fig. 3:**
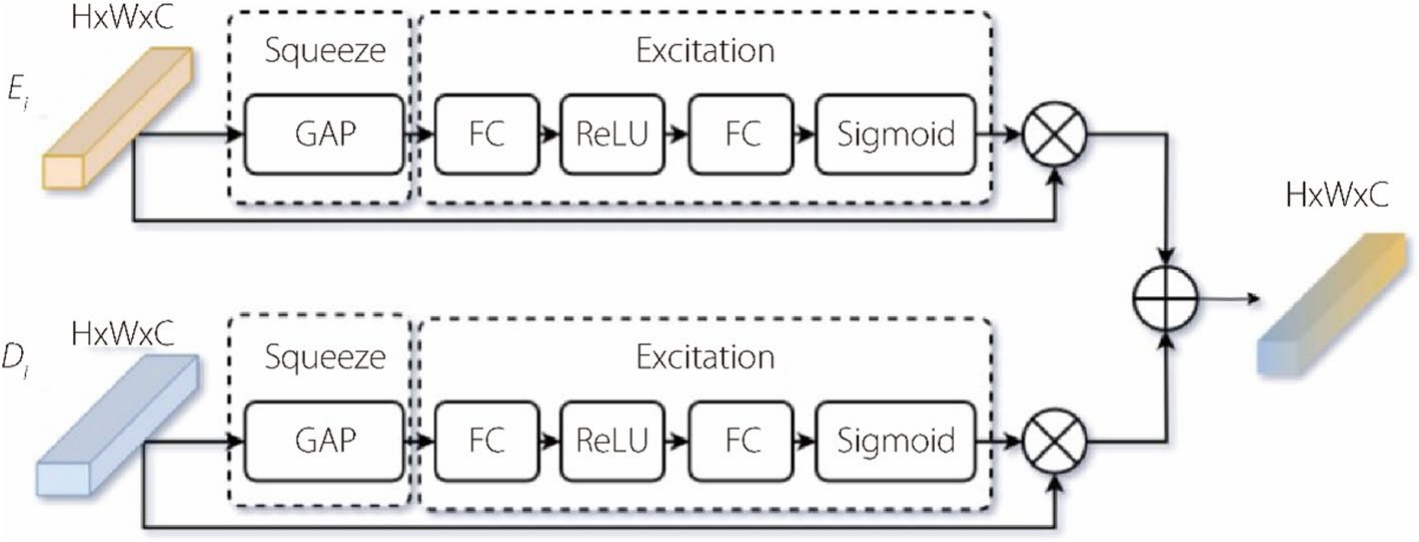
Structure of CAF

To enhance the fusion of the feature maps extracted by the efficientNetV2 and ViTs encoders, we propose a CAF module. The CAF module incorporates an SE block [20] that enables efficient channel attention. The CAF module uses feature maps from both encoders as inputs. First, to ensure compatibility, the dimensions of the feature maps from the efficientNetV2 encoder are adjusted using a 1 × 1 convolutional layer to match the shapes of the feature maps from the ViTs encoder. Next, channel attention is applied to both sets of feature maps. This is achieved by passing the adjusted feature maps from the efficientNetV2 encoder and the feature maps from the ViTs encoder through the SE block. The SE block performs global average pooling on the input feature maps to obtain the global channel descriptors. These descriptors are then passed through two dense layers. The first dense layer reduces the dimensionality of the descriptors by a factor determined by the specified $ration = 8$. The second dense layer applies the sigmoid activation function to generate channel-wise attention weights. The obtained attention weights are multiplied element-wise using their respective feature maps. This process selectively emphasizes the informative features in the feature maps of each encoder, guided by the attention weights. Finally, the fused features are obtained by element-wise addition of the adjusted feature maps from the efficientNetV2 encoder multiplied by the attention weights from the ViTs encoder, and the feature maps from the ViTs encoder multiplied by the attention weights from the adjusted feature maps. This fusion process enables the integration of high-level and local information from both encoders. The CAF module improves the feature fusion process, allowing a more effective combination of complementary information captured by the efficientNetV2 and ViTs encoders. By selectively emphasizing important features and integrating them at the channel level, the CAF module enhances the overall representation and discriminative power of the fused features. By adaptively recalibrating the importance of different channels, the CAF module encourages encoders to focus on the most discriminative and relevant information for the segmentation task. This regularization helps prevent the model from overfitting noisy or irrelevant features, thereby improving generalization. Consequently, it leads to a more accurate BUS segmentation and improves the performance of the multi-task framework. Figure 3 shows the structure of the proposed CAF module.

The combined feature maps using the CAF module serve as the input for the subsequent decoder stage, where the objective is to reconstruct the segmented image. To enhance reconstruction quality, skip connections from the first encoder are incorporated into the decoding process. These skip connections transmit high-resolution spatial information from the first encoder’s earlier layers directly towards the corresponding decoder layers, thereby mitigating the loss of fine-grained details during the upsampling process. The resulting decoder output yields a precise and accurate segmented image, effectively leveraging the strengths of both encoder architectures.

### Classification architecture

Figure 1b shows an overview of the classification of the segmented breast tumor regions extracted from the BUS images. Specifically, we leverage the strengths of the MLP-Mixer [18] model, which is a recently proposed architecture for image classification tasks, to achieve an accurate classification of BUS tumors. The MLP-Mixer model combines MLPs and channel-mixing layers to effectively capture both local and global features in the images. In addition to the promising quality of image classification achieved by this model, to the best of our knowledge, this is the first study that utilizes the MLP-Mixer model for the classification of BUS images. As shown in Fig. 1b, the tumor region is extracted by applying the segmented mask to the image and then resized to $\left(224\times 224\times 3\right)$ to fit the input of the pre-trained MLP-Mixer to obtain the final tumor class.

The architecture of the MLP-Mixer is similar to that of the ViT, where the mixer block takes linearly projected nonoverlapping patches from the input image. Let the input image *x* be of size $\left(H\times W\times C\right)$ and (P, P) be the size of each patch; therefore, the image is split into $S=HW/{P}^{2}$ patches. Furthermore, each patch is linearly projected to form an input vector for the mixer block of size $\left(S\times C\right)$. The mixer is composed of multiple identically sized layers, with each layer comprising two MLP blocks. The first block (token-mixing MLP block) acts on the transpose of *x*, which are the columns of the linear projection table constructed from image X patches. Every row contains the same channel information for all patches. This is fed into a block of two fully connected layers. This block identifies features in the image across patches and aggregates all channels in which the feature occurs. Moreover, the weights are shared in MLP 1. The second block is the channel-mixing MLP block, which operates on the rows after another transpose of *x*. In this phase, computations are applied across all the channels of the patch. This involves searching for features only in that patch and associating it with the channel, whereas in the token-mixing block, it searches for features in all channels. The weights of the MLP blocks are shared across all rows. All the rows of the MLP block share the same weights. Each MLP block includes two fully connected layers, which apply the GELU activation function [48] independently to each row of the input data. Equations 1 and 2 represent the layers of MLP-Mixer:

1 $${U}_{*,i}={X}_{*,i}+{W}_{2}\upsigma \left({W}_{1}LayerNorm{\left(X\right)}_{*,i}\right),\hspace{1em}\hspace{0.25em}i=1\dots C$$

2 $${Y}_{j,*}={U}_{j,*}+{W}_{4}\upsigma \left({W}_{3}LayerNorm{\left(U\right)}_{j,*}\right),\hspace{1em}\hspace{0.25em}j=1\dots S$$

where DS and DC are the tunable hidden widths of the channel-and token-mixing MLPs, respectively. σ denotes the GELU activation function. The computational complexity of the network is linear in the number of input patches *S* owing to the independent selection of DS from the number of patches, preventing it from growing quadratically, unlike ViTs. Furthermore, the model applies the same MLP to every row and column of image *x*. This prevents the model from becoming overly complicated and growing too rapidly when the hidden dimension *C* or sequence length *S* is increased. This approach also results in significant memory saving. In addition to MLP layers, the mixer employs other conventional architectural elements, including skip connections and layer normalization [49]. Following the mixer block, a conventional classification head that includes a global average pooling layer is utilized. This is then succeeded by a linear classifier that produce the final predicted class output.

In addition to employing the MLP-Mixer as a classifier for BUS images, we also investigate the performance of ViT family models. Specifically, we leverage these models to classify BUS images. The ViT model captures global contextual information from the input image by employing the SA mechanism. Specifically, it enables the efficient extraction of relevant features from segmented breast tumor regions.

The ViT’s transformer encoder receives a sequence of flattened, positionally encoded patches of the masked BUS image after resizing it to 224 × 224 × 3. An MSA and an MLP layer make up the transformer encoder module. The MSA layer divides inputs into several heads, allowing each head to learn varying levels of SA. All head outputs are then combined and passed to the MLP to output the final class.

## Results and Discussion

### Datasets

This study involved the assessment of our approach using two publicly available BUS image datasets. Dataset 1 (BUSI) was provided online by Al-Dhabyani et al. [21] and contains 600 BUS images of female patients in a total of 780 PNG format images and their corresponding masks with an average image size of 500px × 500px. The 780 images were split into three classes: normal, benign, and malignant. The benign class had 487 breast tumors, and the malignant class had 210 images. In our work, we only used the malignant and benign. This is because in the first phase, we performed tumor region segmentation and the normal class images did not have any tumor region. Dataset 2 (UDIAT) [50], which was gathered by the Diagnostic Center of the Parc Taul ´ı Corporation, Sabadell (Spain) contains 110 benign and 53 malignant images totaling 163 BUS images and their corresponding masks, which were collected using a Siemens ACUSON Sequoia C512 system 17L5 HD linear array transducer. By evaluating these datasets, we provide a comprehensive analysis of the effectiveness and robustness of the proposed method. The utilization of publicly accessible datasets in our study ensures the reproducibility and generalizability of our findings and allows for comparison with other related methods and techniques.

### Evaluation metrics

To assess the effectiveness of the segmentation models, we employed the following widely used metrics: Dice coefficient (DC), Jaccard index intersection over union (IoU), precision, recall, sensitivity, specificity, and F1-score. By utilizing these common metrics, we could comprehensively evaluate the performance of the segmentation models and compare our results with those of other studies in the field.

3 $$Accuracy=\frac{TP+TN}{TP+TN+FP+FN}$$

4 $$Precision=\frac{TP}{TP+FP}$$

5 $$Recall=\frac{TP}{TP+FN}$$

6 $$Specifity=\frac{TN}{TN+FP}$$

7 $$Sensitivity=\frac{TP}{TP+FP}$$

8 $$F1-score=\frac{2\times Precision\times Recall}{Precision+Recall}$$

9 $$DC=\frac{2\times TP}{2\times TP+FP+FN}$$

10 $$IoU=\frac{TP}{TP+FP+FN}$$

To evaluate the segmentation results, the DC, IoU, accuracy, recall, and precision were used, whereas the metrics accuracy, precision, recall, specificity, F1-score were used to evaluate the classification results.

### Implementation details

To conduct the experiments, we utilized a fivefold cross-validation method for the same dataset partition. During the training process, 80% of the images were used with the remaining 20% for testing. The validation process was performed using 20% of the training data. For the segmentation task, all the images were resized to 256 × 256 pixels. The first encoder, based on the efficientNetV2-L backbone, was used with pre-trained ImageNet [47] weights, and the second encoder, based on MSA, was trained from scratch. Various combinations of learning rates, batch sizes, and epochs were examined. The best results were achieved using the Adam [51] optimizer with an initial learning rate of 1e^−4^, β1 = 0.9, β2 = 0.999 and 1e^−7^, training for 200 epochs with a batch size of eight, and early stopping. A range of data augmentation techniques was employed on the training set, including random rotation and horizontal flips. The selection of the loss function used in our experiments had a notable effect on the outcomes of our study. To overcome the challenge of an imbalanced class distribution in the dataset, we employed a custom segmentation loss function that combined the binary cross-entropy (BCE) and Dice loss. This combined loss function is denoted by LT and is defined as follows:

11 $${L}_{BCE}=-\frac{1}{N}{\sum }_{i=1}^{N}\left[{y}_{i}{\text{log}}\left({p}_{i}\right)+\left(1-{y}_{i}\right){\text{log}}\left(1-{p}_{i}\right)\right]$$

12 $${L}_{Dice}=1-\frac{2{\sum }_{i=1}^{N}{y}_{i}pi+\upepsilon }{{\sum }_{i=1}^{N}\left({y}_{i}+{p}_{i}\right)+\upepsilon }$$

13 $${L}_{T}={L}_{Dice}+{L}_{BCE}$$

**Fig. 4:**
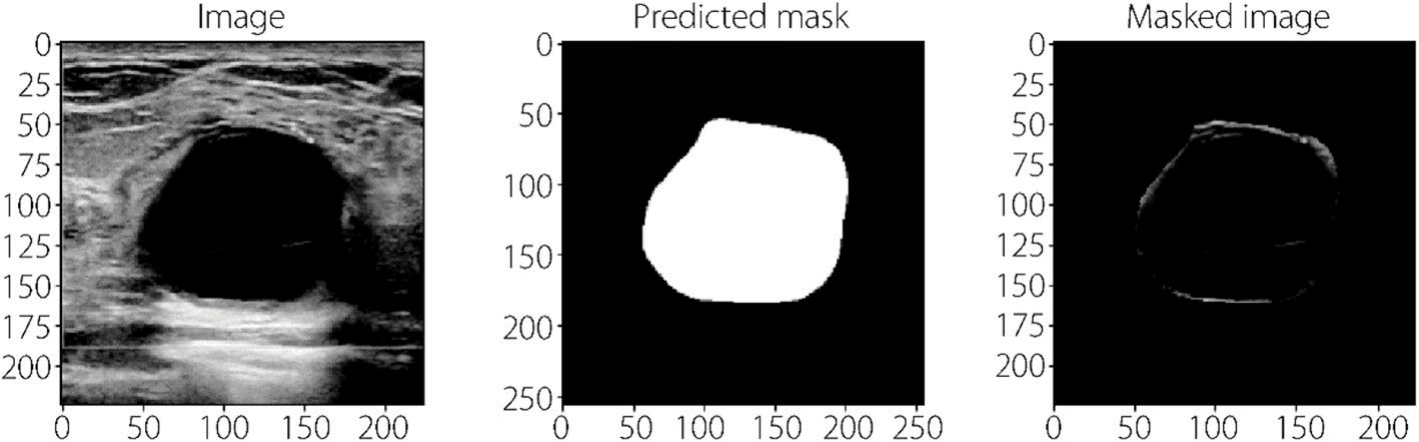
The initial image, the segmentation and the resulted masked image

Using this combined loss function, we were able to account for the class imbalance in the dataset and improve the accuracy and reliability of our segmentation model. As for the classification phase we used two architectures: MLP-Mixer [18] and ViT [13]. The two models take the segmented part of the images that contains only the tumor region and predicts its corresponding class. Figure 4 illustrates an example of the masked images fed to the classification models. The ViT model was trained on masked images resulting from segmentation of the tumor region using the proposed segmentation model. Three versions of ViT model were trained with B/16, B/32, and L/32, using transfer learning. All models were followed by a flattening layer, batch normalization, a dropout of 0.6, a hidden layer of 11 neurons with GELU activation, batch normalization, and an output layer with sigmoid activation. Similarly, we fine-tuned the versions of MLP-Mixer: Mixer-B/16 and Mixer-L/16, with the provided pre-trained weights, where they were followed by the same architecture as ViT’s base model. We used a BCE loss function, and the Adam optimizer with an initial learning rate of 1e^−4^, β1 = 0.9, β2 = 0.999 and epsilon equal to 1e^−7^. The models were trained for 50 epochs with a batch size of eight, and early stopping was enabled.

### BUS segmentation results and discussion

In the ablation study, we evaluated the performance of our segmentation model with and without the CAF module to assess the contribution of the CAF module in improving the segmentation results. Tables 3 and 4 depict the ablation study results for the base model without the CAF module and the proposed method using the CAF module when trained and tested on the BUSI dataset [21], and trained on BUSI dataset [21] and tested on the UDIAT dataset [50] respectively. For the base model without the CAF module, we observed that it achieved competitive segmentation performance with 81.94% in terms of the DC on BUSI dataset, accurately segmenting tumor regions in the BUS images. Similarly, upon integrating the CAF module into the model, we observed a notable improvement in the segmentation results. The CAF module effectively enhanced the feature fusion process with a 1.48% increase in the DC, allowing for better integration of complementary information from the efficientNetV2 and ViTs encoders. We noticed a similar increase in performance when training and testing on the BUSI dataset as well as when training on the BUSI dataset and testing on the UDIAT dataset. This improved feature fusion led to more precise and detailed segmentation boundaries, resulting in enhanced segmentation accuracy and overall performance. The ablation study results demonstrate the significant benefit of incorporating the CAF module, highlighting its effectiveness in improving the segmentation capabilities of our multi-task framework.

**Table 3:** Results of performing ablation study of our proposed method trained and tested on the BUSI [21] dataset

Fold	Accuracy (%)	DC (%)	IoU (%)	Precision (%)	Recall (%)
Baseline	93.860 ± 0.010	81.940 ± 0.004	69.700 ± 0.010	76.820 ± 0.020	87.900 ± 0.010
Baseline + CAF	94.040 ± 0.010	83.420 ± 0.007	72.560 ± 0.010	80.100 ± 0.010	88.100 ± 0.008

**Table 4:** Results of performing ablation study of our proposed method trained on the BUSI [21] dataset and tested on the UDIAT [50] dataset

Fold	Accuracy (%)	DC (%)	IoU (%)	Precision (%)	Recall (%)
Baseline	97.760 ± 0.002	81.440 ± 0.040	70.260 ± 0.010	89.460 ± 0.020	76.400 ± 0.030
Baseline + CAF	97.880 ± 0.000	81.520 ± 0.007	70.320 ± 0.010	90.320 ± 0.020	76.680 ± 0.020

We compared the segmentation results with existing methods. We evaluated our segmentation results mainly using the results found in ref. [52], where a novel approach for the segmentation of BUS images was proposed. Ma et al. [52] introduced a U-shaped architecture called ATFE-Net, which they integrated into an axial-trans (axial transformer) to extract long-range dependencies, and a transformer-based feature enhancement module (trans-FE) was used to capture the reliance between different layers at different depths of the network. In their work, they evaluated their proposed method on the two available BUS datasets: BUSI and UDIAT. Furthermore, we conducted the training process of our suggested segmentation model in accordance with their prescribed methodology, ensuring identical training conditions and fair evaluation for comparative purposes. They evaluated their findings using the following SOTA methods: TransUNet [17], LinkNet [53], D-LinkNet [54], Axial-DeepLab [40], U-Net [8] and UT-Net [55]. Table 5 compares our results with refs. [52] and [56] on the BUSI dataset, whereas Table 6 presents a comparison of the results with ref. [52] when trained on the BUSI dataset and tested on UDIAT dataset.

**Table 5:** Quantitative comparison of segmentation performance with different methods on the BUSI [21] dataset

Model	Accuracy (%)	DC (%)	IoU (%)	Sensitivity (%)
U-Net [8]	95.55	77.19	62.71	74.74
UT-Net [55]	95.58	78.08	64.02	77.93
LinkNet [54]	96.07	81.22	68.21	81.77
TransUNet [17]	96.10	81.57	68.69	82.05
D-LinkNet [54]	96.21	81.72	68.68	82.56
Axial-DeepLab [40]	96.31	82.01	69.00	80.36
ATFE-Net [52]	96.32	82.46	69.73	**82.78**
Ours	**94.04**	**83.42**	**72.56**	80.10

**Table 6:** Quantitative comparison of segmentation performance with different methods trained on the BUSI dataset and tested on UDIAT dataset

Model	Accuracy (%)	DC (%)	IoU (%)	Sensitivity (%)
U-Net [8]	97.08	70.02	54.45	75.57
UT-Net [55]	95.79	58.40	41.84	67.60
LinkNet [54]	96.92	73.55	59.09	89.77
TransUNet [17]	97.61	76.37	62.51	87.28
D-LinkNet [54]	97.66	77.96	64.74	88.00
Axial-DeepLab [40]	97.60	77.16	62.33	84.31
ATFE-Net [52]	97.81	78.44	65.03	85.20
Ours	**97.88**	**81.52**	**70.32**	**90.32**

The quantitative comparison results presented in Table 5 and 6 demonstrate the competitive segmentation performance of the proposed method compared with SOTA methods on the BUSI and UDIAT datasets. Our model achieved DCs of 83.42% and 81.52% when tested on the BUSI and UDIAT datasets respectively, outperforming all other methods in accurately delineating tumor regions. The IoU metric further supported our model’s performance, with scores of 72.56% and 70.32% when tested on BUSI and UDIAT datasets respectively, indicating a substantial overlap between the predicted and ground truth segmentation masks. While our model exhibited a slightly lower accuracy of 94.04% when tested on BUSI dataset compared to other methods, it is important to note that accuracy alone may not fully capture the quality of the segmentation results. Furthermore, our model achieved a sensitivity of 80.10% and 90.32%, effectively capturing the majority of tumor regions. Overall, the proposed method demonstrated competitive performance in segmenting BUSI tumors, with particular emphasis on achieving high-precision and accurate tumor boundary delineation. The incorporation of the CAF module into our model contributed to these improved segmentation results by enhancing the fusion of features and capturing fine details in the tumor regions.

To justify the necessity of using a dual-branch architecture for the proposed method, we conducted a quantitative comparison of the results provided by a U-Net with a pre-trained EfficientNetV2 as a backbone and the proposed method with the dual encoders (EfficientNetV2 and adapted ViTs). The DC is higher for the proposed method (83.42%) than for U-Net with EfficientNetV2-L backbone (81.80%). This indicated that the proposed method accurately captured a larger portion of the tumor region. The IoU coefficient was also higher for the proposed method (72.56%) than for U-Net with EfficientNetV2-L (70.04%). Moreover, the precision and recall values for the proposed method (80.10% and 88.10%, respectively) are higher than those for U-Net with EfficientNetV2-L back-bone (77.62% and 88.04%, respectively). The proposed method achieved an accuracy of 94.04%, whereas U-Net with EfficientNetV2-L achieved a slightly higher accuracy of 96.32%. However, the proposed method outperformed U-Net in terms of the other segmentation performance metrics. Therefore, the proposed method with a dual-branch architecture outperformed U-Net with EfficientNetV2-L backbone in terms of the DC, IoU, and precision, and achieved competitive accuracy. These findings provide additional experimental evidence justifying the necessity of using a dual-branch architecture in the segmentation network. The dual-branch architecture likely enables better capture of both local and global contextual information, leading to more accurate and robust tumor segmentation results in BUS images.

**Fig. 5:**
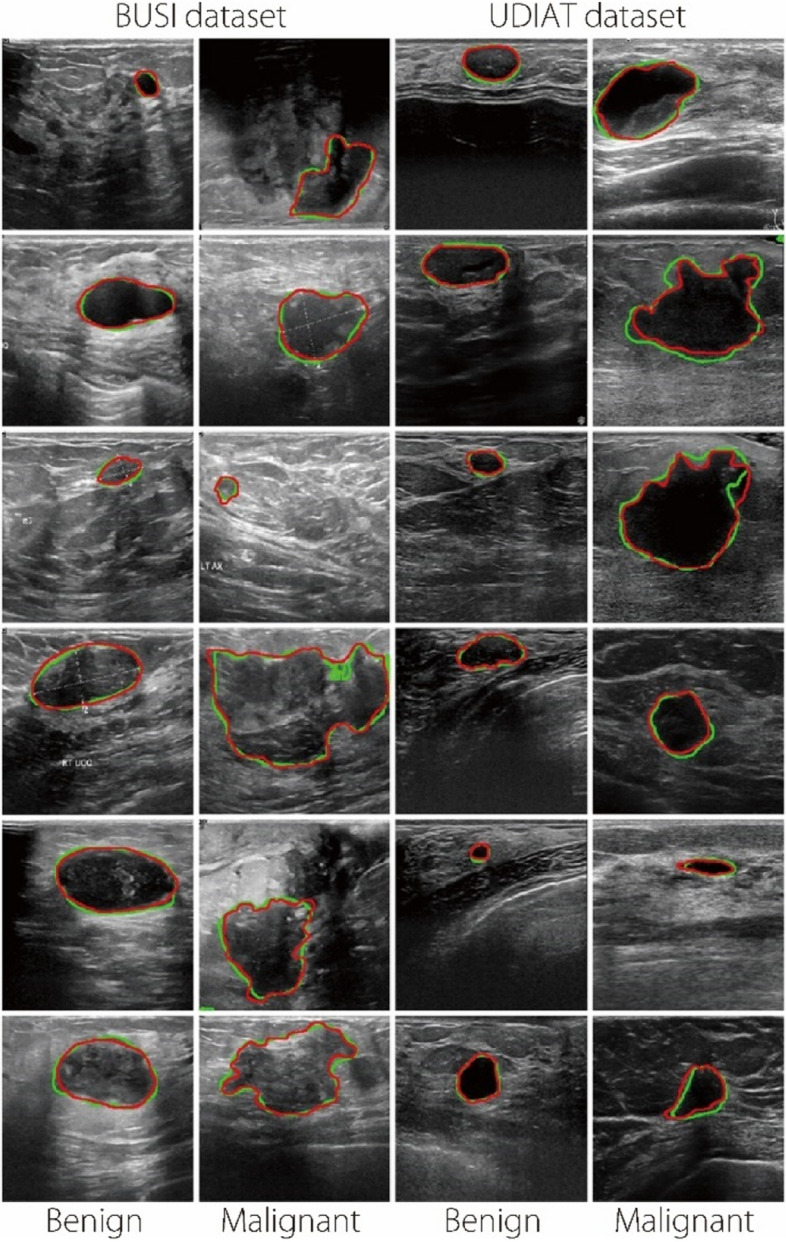
Segmentation examples predicted by our proposed method on BUSI [21] and UDIAT [50] datasets. The red contours represent the segmentation results, while the green contour represents the ground truth

**Fig. 6:**
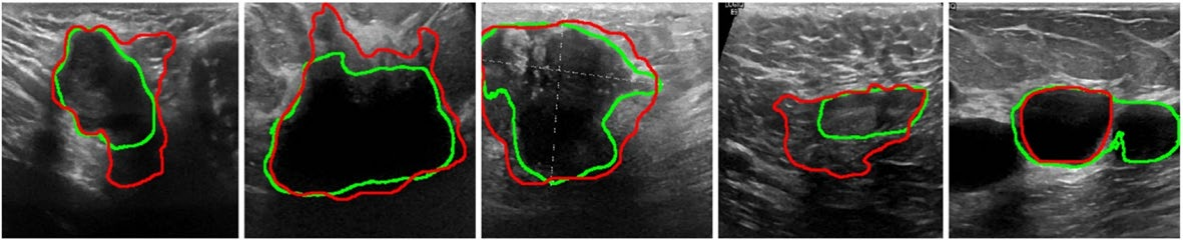
Visualization of some failed cases for segmenting the tumor region of the BUS images by the proposed method. The red contours represent the segmentation results, while the green contour represents the ground truth

Figure 5 shows a visual representation of the segmentation examples. For both Dataset 1 and Dataset 2, the input images with red contours represent the segmentation results, and the green contour represents the actual mask. The model demonstrated an impressive ability to segment both malignant and benign tumors. It is particularly adept at segmenting benign tumors, although it encounters some challenges with malignant tumors. Some challenging tumor examples can be observed in the second row and malignant column of the BUSI dataset, which features a large malignant tumor in the third row with a hidden lesion, and in the fourth row with an irregular shape. The model can segment both large and small tumor regions owing to the incorporation of EfficientNetV2-L features at various blocks with a wide range of scales combined with the global features extracted using the transformer encoder. Figure 5 shows visual examples of the segmentation on the UDIAT dataset [50] using the model trained on the BUSI dataset [21]. These results indicate that the model can be effectively generalized for various datasets. Tumors of varying sizes are recognized and segmented accurately; however, some difficulties arise when handling malignant tumors. Although our proposed network exhibited strong segmentation outcomes, it had limitations in terms of accurately segmenting specific BUS images. Figure 6 illustrates instances of unsuccessful segmentation, emphasizing the difficulties encountered when accurately delineating lesion regions where the boundary is unclear and irregularly shaped, and the lesion region is hidden.

### BUS classification results and discussion

Our study examined the effectiveness of diverse versions of MLP-Mixer and ViTs in classifying the segmented BUS lesions, with the aim of determining their efficacy. Table 7 presents the quantitative results of different models trained on the segmented tumor regions of the BUSI [21] dataset using a fivefold cross validation.

**Table 7:** Classification results reported by the proposed models trained on BUSI [21] dataset

Method	Accuracy (%)	Precision (%)	Recall (%)	F1-score (%)	Sensitivity (%)	Specificity (%)
ViT-B/16	**86.00**	**86.11**	**86.02**	**85.93**	86.45	**85.26**
ViT-B/32	83.61	83.87	83.60	83.56	86.66	78.55
ViT-L/32	85.06	84.40	84.53	84.40	86.87	82.10
MLP-Mixer-B/16	84.13	84.49	84.13	84.09	**89.42**	79.64
MLP-Mixer-L/16	85.46	85.73	85.46	85.47	87.52	82.10

We evaluated the classification results found with the proposed models: ViT and MLP-Mixer and compared it with the classification results reported in ref. [45]. In their research, the authors developed a network, referred to as MsGoF, to classify BUS tumors as malignant or benign. Moreover, they trained their method on three available datasets including the BUSI [21] dataset. A fivefold cross validation was utilized to fully evaluate the effectiveness of the method, with 20% of the training set used as a validation set. Our results were also compared with those reported in ref. [46]. Their work focused on classifying BUS tumors using a modified InceptionV3 network. They introduced an increased number of residual modules and adjusted the hyperparameters of their models. Moreover, we compared our classification results with those reported in ref. [57]. Their study used a combination of supervised and unsupervised learning methods to classify BUS images. To ensure a fair comparison, all the results reported in this section were obtained using the aforementioned methods from the cited studies on the same dataset. Table 8 presents a quantitative comparison of the obtained results with those of previous studies reported in refs. [45] and [46].

**Table 8:** Quantitative comparison with classification models from the literature

Method	Accuracy (%)	Precision (%)	Recall (%)	F1-score (%)	Sensitivity (%)	Specificity (%)
[46]	81.00	83.00	77.00	80.00	-	-
[45]	85.32	-	-	78.96	85.24	**88.57**
URepNet-v1 + SVM (linear) [57]	77.44	68.67	64.38	64.50	-	-
URepNet-v2 + SVM (linear) [57]	77.59	66.30	66.19	65.67	**-**	-
URepNet-v3 + SVM (linear) [57]	80.06	75.47	62.05	65.21	-	-
Ours (ViT-B/16)	**86.00**	**86.11**	**86.02**	**85.93**	86.45	85.26
Ours (MLP-Mixer-B/16)	84.13	84.49	84.13	84.09	**89.42**	79.64

The classification accuracy results demonstrate that the ViT with Base size and 16 × 16 patches (ViT-B/16) achieved the highest accuracy of 86% among the considered models. Although ViT-B/16 has a smaller architecture compared to ViT-B/32 and ViT-L/32, requiring fewer computational resources, it provided slightly better performance. This can be attributed to two factors. Firstly, the larger ViT-L/32 model is a more complex network trained on a substantially larger dataset, which may lead to overfitting, especially for the smaller BUS dataset. Secondly, the smaller patch size of 16 × 16 in ViT-B/16 enables the extraction of more granular features by the transformer encoder compared to the 32 × 32 patches in ViT-L/32. Despite being a data-intensive model, the MLP-Mixer also achieved a competitive accuracy of 85.46% for MLP-Mixer-L/16. This substantiates the significance and effectiveness of MLPs in these architectures.

The results demonstrate that model complexity and patch size are crucial considerations for optimizing the performance of ViT models, especially when limited data is available. For the given dataset, ViT-B/16 provides the optimal balance, yielding the highest accuracy with reasonable computational requirements. The competitive performance of MLP-Mixer-L/16 also highlights the importance of MLPs in extracting local and global features. Furthermore, in the context of medical imaging, it is crucial to lower the false negative rate of a predictive model and augment the true positives rate. The quantitative results of our study demonstrate that, despite having a lower accuracy of 85.46% compared to ViT’s 86.00%, the MLP-Mixer model exhibits superior sensitivity with a score of 89.42%. This indicates that the MLP-Mixer model is more capable of accurately detecting positive cases or true positives and identifying the presence of tumors in BUS images. As a result, MLP-Mixer’s higher sensitivity score leads to improved performance and greater accuracy in predicting tumors in BUS images.

Table 8 presents a comprehensive quantitative comparison of the proposed method and other classification models. Our method achieved an accuracy of 86.00%, outperforming the results reported in ref. [45] (85.32%). In terms of recall, our method achieved a score of 86.02%, surpassing the results reported in ref. [45] (77.00%) and URepNet-v3 + SVM (linear) (62.05%). The F1-score for our method was 85.93%, which is higher than the values reported in ref. [44] (78.96%) and URepNet-v3 + SVM (linear) (65.21%). Although the sensitivity and specificity scores were not reported in previous studies [46, 57], our method exhibited a sensitivity of 86.45%, indicating its ability to correctly identify positive samples, and a specificity of 85.26%, highlighting its capability to correctly classify negative samples. However, the method reported in ref. [45] achieved a better specificity recording 88.57%, which might be explained by the fact that the authors may have employed specific strategies or features in their model design that are particularly effective in distinguishing non-lesion regions, or by the choice of evaluation metrics and thresholds that can impact specificity. Our method outperformed other models in terms of accuracy, precision, recall, and F1-score, suggesting its effectiveness for BUS classification. Notably, our method demonstrated competitive results when using two different backbone architectures, ViT-B/16 and MLP-Mixer-B/16, with accuracies of 86.00% and 84.13%, respectively. These findings validate the superiority of our proposed approach in accurately classifying breast lesions and showcase the potential of both ViT and MLP-Mixer architectures for this task. Our method, which utilizes MLP-Mixer and ViT architectures to classify the overlay of the generated segmentation over the original images, achieved superior results owing to several key factors. First, both MLP-Mixer and ViT are powerful neural network architectures that have demonstrated excellent performance in various computer vision tasks. These architectures excel at capturing intricate patterns and relationships in images, enabling them to effectively analyze the overlay of the generated segmentation and original images. Second, MLP-Mixer and ViT architectures can capture global contextual information from the entire image. This global perspective helps capture important features and contexts that contribute to accurate classification. Additionally, the classification of the overlay of the generated mask on the original images serves as a valuable visual cue for classification, where the network, while fine-tuning, focuses solely on the tumor regions.

One limitation of our current work is the potential oversight of the tumor’s surrounding environment in the diagnostic process. Although our multi-task framework focuses on accurate tumor segmentation and classification, it does not explicitly incorporate contextual information regarding the tumor’s immediate surroundings. This omission may hinder further improvements in classification accuracy, as the surrounding environment can provide valuable insights for diagnosis. We acknowledge the importance of considering this aspect and will address it in future studies. Future research endeavors will explore methods that explicitly model the tumor’s surrounding environment to enhance the classification accuracy and provide a more comprehensive understanding of the tumor’s diagnostic characteristics. By incorporating this contextual information, we aim to further improve the accuracy and reliability of the proposed method.

## Conclusions

In conclusion, this study explored a novel hybrid method for the segmentation and classification of breast tumors in BUS images by leveraging the capabilities of CNNs, attention mechanisms, and MLPs. The framework utilizes a two-encoder architecture that incorporates an EfficientNetV2 backbone and a customized ViT encoder to effectively extract tumor regions from BUS images. The SA mechanism of the transformer encoder enables the capture of a broad range of high-level and complex features, whereas the EfficientNetV2 encoder preserves the local information within the images. To fuse these extracted features, a CAF module was introduced, selectively emphasizing important features from both encoders. The integration of high-level and local information results in improved feature integration. The feature maps obtained were subsequently reconstructed using a decoder to generate segmentation maps that effectively delineated the tumor regions. Furthermore, the proposed method incorporated a novel approach for lesion classification in BUS images, employing an MLP-Mixer-based classifier, which, to the best of our knowledge, has been applied for the first time in this specific task. The classification results demonstrate the effectiveness of the proposed approach, achieving an accuracy of 86.00%. The experimental evaluation shows the superior performance of the proposed framework compared with recent related works. The segmentation results exhibited an impressive DC of 83.42%, indicating highly accurate tumor region delineation. In addition, the classification accuracy of 86% further supports the superiority of the proposed method. These findings highlight the potential of ViTs and MLP-Mixer architectures in medical image analysis, specifically for BUS image segmentation and classification tasks. The proposed multi-task framework, which incorporates the hybrid architecture and CAF module, effectively integrates high-level and local information, leading to improved segmentation and classification results. This study contributes to advancing the field of medical image analysis by introducing a novel and efficient approach that outperforms existing methods in terms of both segmentation and classification.

## Data Availability

The public datasets used in this study are publicly available BUSI provided online [21] as well as UDIAT dataset [50].

## Abbreviations

CNN: Convolutional neural network
ViT: Vision transformer
MLP: Multilayer perceptron
SA: Self-attention
CAF: Channel attention fusion
BUS: Breast ultrasound
SOTA: State of the art
NLP: Natural language processing
ROI: Regions of interest
SE: Squeeze-and-excitation
CAD: Computer-aided diagnosis
GAN: Generative adversarial network
SCA: Self-co-attention
GELU: Gaussian error liinear unit
MSA: Multi-head-self-attention
IoU: Intersection over union
DC: Dice coefficient
BCE: Binary cross-entropy

## References

[1] Siegel RL, Miller KD, Jemal A (2018). Cancer statistics, 2018. CA Cancer J Clin..

[2] Bray F, Ferlay J, Soerjomataram I, Siegel RL, Torre LA, Jemal A (2018). Global cancer statistics 2018: GLOBOCAN estimates of incidence and mortality worldwide for 36 cancers in 185 countries. CA Cancer J Clin.

[3] Zhang HY, Meng ZL, Ru JY, Meng YQ, Wang K (2023). Application and prospects of AI-based radiomics in ultrasound diagnosis. Vis Comput Ind Biomed Art.

[4] Sippel S, Muruganandan K, Levine A, Shah S (2011). Review article: use of ultrasound in the developing world. Int J Emerg Med.

[5] Barra S, Carta SM, Corriga A, Podda AS, Recupero DR (2020). Deep learning and time series-to-image encoding for financial forecasting. IEEE/CAA J Autom Sin.

[6] Piccialli F, Somma VD, Giampaolo F, Cuomo S, Fortino G (2021). A survey on deep learning in medicine: why, how and when?. Inf Fusion.

[7] Le EPV, Wang Y, Huang Y, Hickman S, Gilbert FJ (2019). Artificial intelligence in breast imaging. Clin Radiol.

[8] Ronneberger O, Fischer P, Brox T (2015) U-Net: convolutional networks for biomedical image segmentation. In: Navab N, Hornegger J, Wells WM, Frangi AF (eds) Medical image computing and computer-assisted intervention. 18th international conference, Munich, October 2015. Lecture notes in computer science (Image processing, computer vision, pattern recognition, and graphics), vol 9351. Springer, Heidelberg, pp 234-241. 10.1007/978-3-319-24574-4_28

[9] Hu H, Zhang Z, Xie ZD, Lin S (2019) Local relation networks for image recognition. In: Proceedings of 2019 IEEE/CVF international conference on computer vision, IEEE, Seoul, 27 October-2 November 2019. 10.1109/ICCV.2019.00356

[10] Vaswani A, Shazeer N, Parmar N, Uszkoreit J, Jones L, Gomez AN et al (2017) Attention is all you need. In: Proceedings of the 31st international conference on neural information processing systems, Curran Associates, Inc., Long Beach, 4-9 December 2017

[11] Han K, Wang YH, Chen HT, Chen XH, Guo JY, Liu ZH (2023). A survey on vision transformer. IEEE Trans Pattern Anal Mach Intell.

[12] Al-hammuri K, Gebali F, Kanan A, Chelvan IT (2023). Vision transformer architecture and applications in digital health: a tutorial and survey. Vis Comput Ind Biomed Art.

[13] Dosovitskiy A, Beyer L, Kolesnikov A, Weissenborn D, Zhai XH, Unterthiner T et al (2021) An image is worth 16 × 16 words: transformers for image recognition at scale. In: Proceedings of the 9th international conference on learning representations, ICLR, Vienna, 3-7 May 2021

[14] Li J, Chen JY, Tang YC, Wang C, Landman BA, Zhou SK (2023). Transforming medical imaging with transformers? A comparative review of key properties, current progresses, and future perspectives. Med Image Anal.

[15] Cao H, Wang YY, Chen J, Jiang DS, Zhang XP, Tian Q et al (2023) Swin-Unet: Unet-like pure transformer for medical image segmentation. In: Karlinsky L, Michaeli T, Nishino K (eds) Computer vision - ECCV 2022 workshops. ECCV 2022. Lecture notes in computer science, vol 13803. Springer, Cham, pp 205-218. 10.1007/978-3-031-25066-8_9

[16] Azad R, Kazerouni A, Heidari M, Aghdam EK, Molaei A, Jia YW (2024). Advances in medical image analysis with vision transformers: a comprehensive review. Med Image Anal.

[17] Chen JN, Lu YY, Yu QH, Luo XD, Adeli E, Wang Y et al (2021) TransUNet: transformers make strong encoders for medical image segmentation. arXiv preprint arXiv: 2102.04306

[18] Tolstikhin IO, Houlsby N, Kolesnikov A, Beyer L, Zhai XH, Unterthiner T et al (2021) MLP-mixer: an all-MLP architecture for vision. In: Proceedings of the 34th international conference on neural information processing systems, NeurIPS, Online, 6-14 December 2021

[19] Tan MX, Le Q (2021) EfficientNetV2: smaller models and faster training. In: Proceedings of the 38th international conference on machine learning, ICML, Online, 18-24 July 2021

[20] Hu J, Shen L, Sun G (2018) Squeeze-and-excitation networks. In: Proceedings of 2018 IEEE/CVF conference on computer vision and pattern recognition, IEEE, Salt Lake City, 18-23 June 2018. 10.1109/CVPR.2018.00745

[21] Al-Dhabyani W, Gomaa M, Khaled H, Fahmy A (2020). Dataset of breast ultrasound images. Data Brief.

[22] Vigil N, Barry M, Amini A, Akhloufi M, Maldague XPV, Ma L (2022). Dual-intended deep learning model for breast cancer diagnosis in ultrasound imaging. Cancers.

[23] Xing J, Li ZR, Wang BY, Qi YJ, Yu BB, Zanjani FG (2021). Lesion segmentation in ultrasound using semi-pixel-wise cycle generative adversarial nets. IEEE/ACM Trans Comput Biol Bioinform.

[24] He KM, Zhang XY, Ren SQ, Sun J (2016) Deep residual learning for image recognition. In: Proceedings of 2016 IEEE conference on computer vision and pattern recognition, IEEE, Las Vegas, 27-30 June 2016. 10.1109/CVPR.2016.90

[25] Singh VK, Abdel-Nasser M, Akram F, Rashwan HA, Sarker MMK, Pandey N (2020). Breast tumor segmentation in ultrasound images using contextual-information-aware deep adversarial learning framework. Expert Syst Appl.

[26] Lei BY, Huang S, Li R, Bian C, Li H, Chou YH (2018). Segmentation of breast anatomy for automated whole breast ultrasound images with boundary regularized convolutional encoder-decoder network. Neurocomputing.

[27] Lei BY, Huang S, Li H, Li R, Bian C, Chou YH (2020). Self-co-attention neural network for anatomy segmentation in whole breast ultrasound. Med Image Anal.

[28] Kumar V, Webb JM, Gregory A, Denis M, Meixner DD, Bayat M (2018). Automated and real-time segmentation of suspicious breast masses using convolutional neural network. PLoS One.

[29] Vakanski A, Xian M, Freer PE (2020). Attention-enriched deep learning model for breast tumor segmentation in ultrasound images. Ultrasound Med Biol.

[30] Tong Y, Liu YY, Zhao MX, Meng L, Zhang JC (2021). Improved U-net MALF model for lesion segmentation in breast ultrasound images. Biomed Signal Process Control.

[31] Yang HN, Yang DP (2023). CSwin-PNet: a CNN-Swin transformer combined pyramid network for breast lesion segmentation in ultrasound images. Expert Syst Appl.

[32] Al-Battal AF, Lerman IR, Nguyen TQ (2023). Multi-path decoder U-Net: a weakly trained real-time segmentation network for object detection and localization in ultrasound scans. Comput Med Imaging Graph.

[33] Farooq MU, Ullah Z, Gwak J (2023). Residual attention based uncertainty-guided mean teacher model for semi-supervised breast masses segmentation in 2D ultrasonography. Comput Med Imaging Graph.

[34] Moon WK, Shen YW, Huang CS, Chiang LR, Chang RF (2011). Computer-aided diagnosis for the classification of breast masses in automated whole breast ultrasound images. Ultrasound Med Biol.

[35] Flores WG, Pereira WCDA, Infantosi AFC (2015). Improving classification performance of breast lesions on ultrasonography. Pattern Recognit.

[36] Gómez W, Rodríguez A, Pereira WCA, Infantosi AFC (2013) Feature selection and classifier performance in computer-aided diagnosis for breast ultrasound. In: Proceedings of the 2013 10th international conference and expo on emerging technologies for a smarter world, IEEE, Melville, 21-22 October 2013. 10.1109/CEWIT.2013.6713755

[37] Tanaka H, Chiu SW, Watanabe T, Kaoku S, Yamaguchi T (2019). Computer-aided diagnosis system for breast ultrasound images using deep learning. Phys Med Biol.

[38] Han S, Kang HK, Jeong JY, Park MH, Kim W, Bang WC (2017). A deep learning framework for supporting the classification of breast lesions in ultrasound images. Phys Med Biol.

[39] Szegedy C, Liu W, Jia YQ, Sermanet P, Reed S, Anguelov D et al (2015) Going deeper with convolutions. In: Proceedings of 2015 IEEE conference on computer vision and pattern recognition, IEEE, Boston, 7-12 June 2015. 10.1109/CVPR.2015.7298594

[40] Wang Y, Choi EJ, Choi Y, Zhang H, Jin GY, Ko SB (2020). Breast cancer classification in automated breast ultrasound using multiview convolutional neural network with transfer learning. Ultrasound Med Biol.

[41] Byra M, Galperin M, Ojeda-Fournier H, Olson L, O’Boyle M, Comstock C (2019). Breast mass classification in sonography with transfer learning using a deep convolutional neural network and color conversion. Med Phys.

[42] Xiao T, Liu L, Li K, Qin WJ, Yu SD, Li ZC (2018). Comparison of transferred deep neural networks in ultrasonic breast masses discrimination. BioMed Res Int.

[43] Ayana G, Choe SW (2022). Buvitnet: breast ultrasound detection via vision transformers. Diagnostics.

[44] Lu SY, Wang SH, Zhang YD (2022). SAFNet: a deep spatial attention network with classifier fusion for breast cancer detection. Comput Biol Med.

[45] Zhong SZ, Tu C, Dong XY, Feng QJ, Chen WF, Zhang Y (2023). MsGoF: breast lesion classification on ultrasound images by multi-scale gradational-order fusion framework. Comput Methods Programs Biomed.

[46] Sirjani N, Oghli MG, Tarzamni MK, Gity M, Shabanzadeh A, Ghaderi P (2023). A novel deep learning model for breast lesion classification using ultrasound images: a multicenter data evaluation. Phys Med.

[47] Russakovsky O, Deng J, Su H, Krause J, Satheesh S, Ma SA (2015). ImageNet large scale visual recognition challenge. Int J Comput Vis.

[48] Hendrycks D, Gimpel K (2016) Gaussian error linear units (GELUs). arXiv preprint arXiv: 1606.08415

[49] Ba JL, Kiros JR, Hinton GE (2016) Layer normalization. arXiv preprint arXiv: 1607.06450

[50] Yap MH, Pons G, Martí J, Ganau S, Sentís M, Zwiggelaar R (2018). Automated breast ultrasound lesions detection using convolutional neural networks. IEEE J Biomed Health Inform.

[51] Kingma DP, Ba J (2015) Adam: a method for stochastic optimization. In: Proceedings of the 3rd International conference on learning representations, ICLR, San Diego, 7-9 May 2015

[52] Ma Z, Qi YL, Xu CB, Zhao W, Lou M, Wang YM (2023). ATFE-Net: axial transformer and feature enhancement-based CNN for ultrasound breast mass segmentation. Comput Biol Med.

[53] Chaurasia A, Culurciello E (2017) LinkNet: Exploiting encoder representations for efficient semantic segmentation. In: Proceedings of 2017 IEEE visual communications and image processing, IEEE, St. Petersburg, 10-13 December 2017. 10.1109/VCIP.2017.8305148

[54] Zhou ZW, Siddiquee MMR, Tajbakhsh N, Liang JM (2018) UNet++: a nested U-Net architecture for medical image segmentation. In: Stoyanov D, Taylor Z, Carneiro G, Syeda-Mahmood T, Martel A, Maier-Hein L et al (eds) Deep learning in medical image analysis and multimodal learning for clinical decision support. DLMIA ML-CDS 2018 2018. Lecture Notes in Computer Science(), vol 11045. Springer, Cham, pp 3-11. 10.1007/978-3-030-00889-5_1 10.1007/978-3-030-00889-5_1 PMC7329239 32613207

[55] Gao YH, Zhou M, Metaxas DN (2021) UTNet: a hybrid transformer architecture for medical image segmentation. In: de Bruijne M, Cattin PC, Cotin S, Padoy N, Speidel S, Zheng YF et al (eds) Medical image computing and computer assisted intervention–MICCAI 2021. MICCAI 2021. Lecture Notes in Computer Science(), vol 12903. Springer, Cham, pp 61-71. 10.1007/978-3-030-87199-4_6

[56] He QQ, Yang QJ, Xie MH (2023). HCTNet: a hybrid CNN-transformer network for breast ultrasound image segmentation. Comput Biol Med.

[57] Song M, Kim Y (2024). Optimizing proportional balance between supervised and unsupervised features for ultrasound breast lesion classification. Biomed Signal Process Control.

