# Correction: Multi-task approach based on combined CNN-transformer for efficient segmentation and classification of breast tumors in ultrasound images

**DOI:** 10.1186/s42492-024-00156-9

**Published:** 2024-02-09

**Authors:** Jaouad Tagnamas, Hiba Ramadan, Ali Yahyaouy, Hamid Tairi

**Affiliations:** Department of Informatics, Faculty of Sciences Dhar El Mahraz, University of Sidi Mohamed Ben Abdellah, 30000 Fez, Morocco

**Correction** **:** ***Vis. Comput. Ind. Biomed. Art 7, 2 (2024)***

**https://doi.org/10.1186/s42492-024-00155-w**

Following publication of the original article [1], the authors reported that the wrong version of abstract and keywords were mistakenly inserted to this article.

The original Abstract and Keywords were:

Accurate segmentation of breast ultrasound (BUS) images is crucial for early diagnosis and treatment of breast cancer. Further, the task of segmenting lesions in BUS images continues to pose significant challenges due to the limitations of convolutional neural networks (CNNs) in capturing long-range dependencies and obtaining global context information. Existing methods relying solely on CNNs have struggled to address these issues. Recently, ConvNeXts have emerged as a promising architecture for CNNs, while transformers have demonstrated outstanding performance in diverse computer vision tasks, including the analysis of medical images. In this paper, we propose a novel breast lesion segmentation network CS-Net that combines the strengths of ConvNeXt and Swin Transformer models to enhance the performance of the U-Net architecture. Our network operates on BUS images and adopts an end-to-end approach to perform segmentation. To address the limitations of CNNs, we design a hybrid encoder that incorporates modified ConvNeXt convolutions and Swin Transformer. Furthermore, to enhance capturing the spatial and channel attention in feature maps we incorporate the Coordinate Attention Module. Second, we design an Encoder-Decoder Features Fusion Module that facilitates the fusion of low-level features from the encoder with high-level semantic features from the decoder during the image reconstruction. Experimental results demonstrate the superiority of our network over state-of-the-art image segmentation methods for BUS lesions segmentation.

Keywords - Breast ultrasound segmentation, Convolutional neural networks, Swin Transformer, ConvNeXt, Efficient channel attention, Coordinate attention module

The correct Abstract and Keywords should read:

Nowadays, inspired by the great success of Transformers in Natural Language Processing, many applications of Vision Transformers (ViTs) have been investigated in the field of medical image analysis including breast ultrasound (BUS) image segmentation and classification. In this paper, we propose an efficient multi-task framework to segment and classify tumors in BUS images using hybrid convolutional neural networks (CNNs)-ViTs architecture and Multi-Perceptron (MLP)-Mixer. The proposed method uses a two-encoder architecture with EfficientNetV2 backbone and an adapted ViT encoder to extract tumor regions in BUS images. The self-attention (SA) mechanism in the Transformer encoder allows capturing a wide range of high-level and complex features while the EfficientNetV2 encoder preserves local information in image. To fusion the extracted features, a Channel Attention Fusion (CAF) module is introduced. The CAF module selectively emphasizes important features from both encoders, improving the integration of high-level and local information. The resulting feature maps are reconstructed to obtain the segmentation maps using a decoder. Then, our method classifies the segmented tumor regions into benign and malignant using a simple and efficient classifier based on MLP-Mixer, that is applied for the first time, to the best of our knowledge, for the task of lesion classification in BUS images. Experimental results illustrate the outperformance of our framework compared to recent works for the task of segmentation by producing 83.42% in terms of Dice coefficient as well as for the classification with 86% in terms of accuracy.

Keywords - Breast Ultrasound segmentation and classification, Breast tumors, Convolutional Neural Networks, Self-Attention, MLP-Mixer, Channel Attention.

The original article [1] has been updated.
